# Epileptogenic Brain Malformations and Mutations in Tubulin Genes: A Case Report and Review of the Literature

**DOI:** 10.3390/ijms18112273

**Published:** 2017-10-29

**Authors:** Annalisa Mencarelli, Paolo Prontera, Gabriela Stangoni, Elisabetta Mencaroni, Nicola Principi, Susanna Esposito

**Affiliations:** 1 Pediatric Clinic, Department of Surgical and Biomedical Sciences, Università degli Studi di Perugia, 06123 Perugia, Italy; almiomedico@gmail.com (A.M.); elisabetta.mencaroni@ospedale.perugia.it (E.M.); 2 Medical Genetics Unit, S. Maria della Misericordia Hospital, and University of Perugia, 06123 Perugia, Italy; paolo.prontera@ospedale.perugia.it (P.P.); gabriela.stangoni@ospedale.perugia.it (G.S.); 3 Pediatric Highly Intensive Care Unit, Università degli Studi di Milano, Fondazione IRCCS Ca’ Granda Ospedale Maggiore Policlinico, 20122 Milano, Italy; nicola.principi@unimi.it

**Keywords:** malformations of cortical developmental, lissencephaly, epilepsy, *TUBA1A*, neuronal migration disorders

## Abstract

Malformations of the cerebral cortex are an important cause of developmental disabilities and epilepsy. Neurological disorders caused by abnormal neuronal migration have been observed to occur with mutations in tubulin genes. The α- and β-tubulin genes encode cytoskeletal proteins, which play a role in the developing brain. TUBA1A mutations are associated with a wide spectrum of neurological problems, which are characterized by peculiar clinical details and neuroradiologic patterns. This manuscript describes the case of a nine-year-old girl with microcephaly, mild facial dysmorphisms, epileptic seizures, and severe developmental delay, with a de novo heterozygous c.320A>G [p.(His 107 Arg)] mutation in *TUBA1A* gene, and the clinical aspects and neuroimaging features of “lissencephaly syndrome” are summarized. This case shows that *TUBA1A* mutations lead to a variety of brain malformations ranging from lissencephaly with perisylvian pachygyria to diffuse posteriorly predominant pachygyria, combined with internal capsule dysgenesis, cerebellar dysplasia, and callosal hypotrophy. This peculiar neuroradiological pattern, in combination with the usually severe clinical presentation, suggests the need for future molecular studies to address the mechanisms of *TUBA1A* mutation-induced neuropathology.

## 1. Introduction

The term malformation of cortical development describes a group of disorders that are associated with several neurologic problems, including neurodevelopment delay and epilepsy. According to the stage at which the neurodevelopmental process is disrupted, these disorders are divided into three groups: cases due to abnormal cell proliferation and apoptosis, those secondary to abnormal cell migration, and those that depend on abnormal postmigrational development [1]. Mutations of genes that are involved in neurogenesis, cell replication, and neuronal migration are associated with these clinical conditions. Among them, a significant role is played by genes that encode proteins involved in the formation of microtubules (i.e., *TUBA 1A*, *TUBB 2B*, *TUB B3*, *TUB A8*, *TUB G1*) [2,3,4] and genes associated with the synthesis of microtube-associated proteins (i.e., LIS1, DCX, DYNC1H, KIF5C, NDE) [5,6]. Microtubules are, alongside microfilaments and intermediate filaments, the principal components of the cytoskeleton and are involved in nucleic and cell division, organization of intracellular structure and intracellular transport, axonal pathfinding, and ciliary and flagellar motility [5,6]. They are composed of alternating heterodimers of α- and β-tubulin. Mutations in α/β-tubulin genes might alter the dynamic functions of microtubules and cause a wide spectrum of cerebral malformations, including microcephaly, lissencephaly, pachygyria, band heterotopia, abnormal white matter tracts and cranial nerves, and malformations of the mid- and hindbrain [7]. 

Although experimental and clinical data indicate the critical role of microtubules during neural development, the relationship between the clinical phenotype and the mutations in the genes involved in the synthesis of this protein is not precisely defined. Moreover, it has not been clarified whether different mutations in the same gene can lead to different clinical manifestations. For the *TUBA1A* gene, case reports have described that although cortical malformation resembling classical lissencephaly could be detected in all patients with mutations of this gene, specific combinations of features could be identified depending on the particular genetic alteration that is present [8,9,10]. Consequently, the description of additional patients with mutations in the *TUBA1A* gene is essential to better define the spectrum of phenotypes associated with *TUBA1A* mutation. This manuscript presents a patient with a de novo heterozygous *TUBA1A* mutation and summarizes the clinical aspects and neuroimaging features of “lissencephaly syndrome”.

## 2. Materials and Methods

### 2.1. Case Report

**Figure 1:**
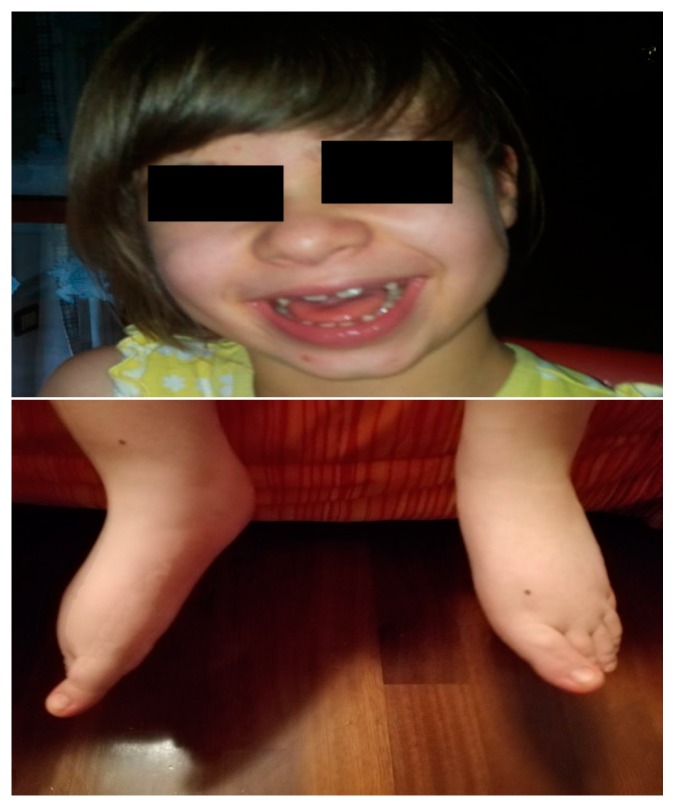
Dysmorphic features of the patient. Pedal edema, microcephaly, bulbous nasal tip, large mouth.

Our patient, a nine-year-old girl, was born at a gestational age of 39 weeks + 6 days. Her birth weight was 3640 gr (75th percentile). Her Apgar score was 10/10 (1 min/5 min). At 28 weeks of gestation, ventricular dilatation was detected by ultrasonography. Screening for toxoplasma, rubella, and cytomegalovirus infections was negative. Her parents were healthy and unrelated. Her older brother was also healthy and had normal development. At one month of age, she began suffering from epileptic seizures and developmental delay, palpebral clonus, and deviation of the eye and the head on the left side. Examination revealed a hypotonic baby, microcephaly (head circumference was 41.2 cm, <3rd percentile) and mild facial dysmorphisms: bulbous nasal tip, large mouth, edema of the hands and feet with camptodactyly, bilateral thelarche, hypoplasia of labia minora, and poor visual and social interactions (Figure 1).

Initial investigation included blood cell count (white blood cells: 4900/µL; red blood cells: 3,770,000/µL; Hb: 11.8 g/dL; platelets: 375,000/µL), liver function tests (SGOT: 35 U/L; SGPT: 38 U/L), serum electrolyte concentration (Na: 135 mEq/L; K: 48 mEq/L; Cl 102 mEq/L; Ca: 5.10 mEq/L), thyroid function (TSH 3.8 mIU/L), metabolic tests and urine organ acid determination, all of which were within normal limits. Electroencephalography demonstrated irregular organization of the background activity and slow waves posteriorly on the left side. Conventional karyotyping revealed a normal female with 46 chromosomes, XX; a DNA screen for Rett Syndrome was negative. At three months of age, the patient suffered from falls due to epileptic fits with severe intellectual disability, and she was started on carbamazepine. 

**Figure 2:**
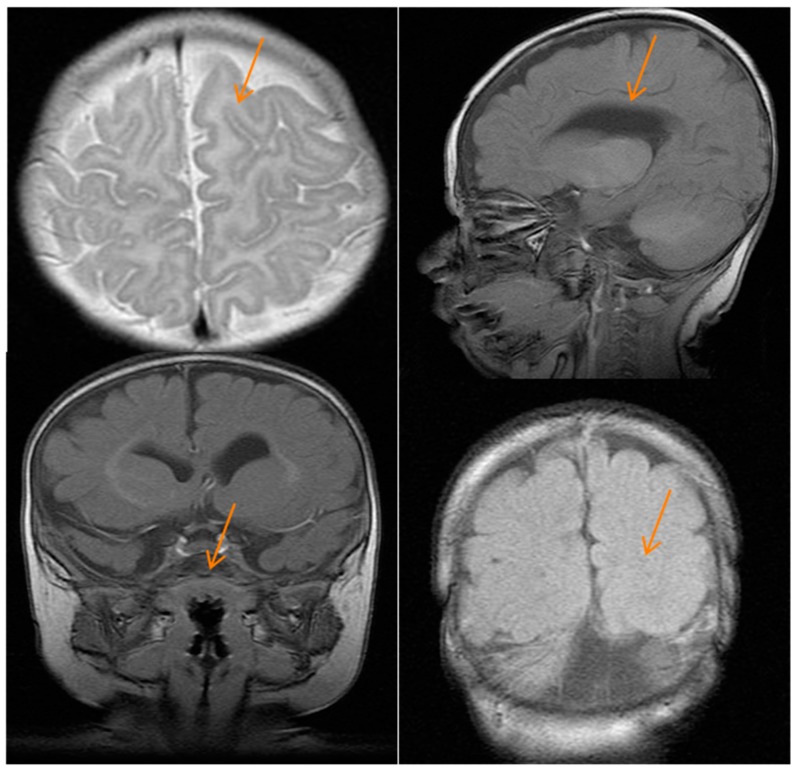
Brain magnetic resonance imaging of the patient. Images show cortical dysgenesis (**top left** and **lower right** figures), a thin corpus callosum (**top right** figure), and hypoplastic pons (**lower left** figure); the red arrows indicate the brain defects.

Brain magnetic resonance imaging at four months showed mild asymmetry and dilatation of lateral ventricles (>in right ventricles) with decreased thickness of the white matter of the same cerebral hemisphere and cortical dysgenesis with dysmorphic frontal lobes, a simplified gyral pattern, and poor development of the sylvian fissure. The corpus callosum was thin, and basal ganglia were hypoplastic. The right caudate nucleus and right lentiform nucleus were dysmorphic. Hypoplasia of cerebellar vermis and pons were also seen (Figure 2). 

Epilepsy was poorly controlled on carbamazepine therapy, so she was treated with phenobarbital with satisfactory seizure control.

### 2.2. Genetic Analyses

Clinical information and blood/DNA samples were obtained after the approval of the Ethics Committee of Umbria Region (22 January 2017) and signed written informed consent. DNA was extracted from peripheral blood leukocytes of the proband using a QiaSymphony SP robot (Qiagen, Hilden, Germany) according to the manufacturer’s protocol. High-quality DNA was quantified using a Quantifluor Fluorometer (Promega, Madison, WI, USA).

The Haloplex panel was designed using the Agilent SureDesign tool (Available online: https://earray.chem.agilent.com/suredesign/index.htm) to capture the 86 epilepsy genes (complete list is available in Supplementary Table S1), as previously described [11].

Variants were annotated with gene name and classified according to their position and effect (frameshift, truncating, splicing, coding non-synonymous, coding synonymous, intronic) using the ANNOVAR tool [12]. Variants reported in the Exome Aggregation Consortium (ExAC) database (http://exac.broadinstitute.org/) and/or in the 1000 Genomes Project (Available online: http://www.1000genomes.org) and/or in the NHLBI Exome Sequencing Project (ESP, ESP6500 database, available online: http://evs.gs.washington.edu/EVS), with a minor allele frequency >0.01 (1%), were dropped. In silico prediction of mutations’ pathogenicity was obtained using the MutationTaster, SNPdryad, and PolyPhen2 databases (Hong Kong, China). 

Putative causative variants were analyzed by Sanger sequencing to confirm the next-generation sequencing results and were investigated in the parents of probands to check their inheritance status. Genomic DNA was extracted from patient and parents peripheral blood using standard protocols using a Qiagen Puregene Blood Core Kit. We performed confirmation of constitutional mutations by direct Sanger sequencing. Polymerase chain reaction (PCR) amplification was performed with 50 ng of genomic DNA using Taq DNA polymerase (Applied Biosystems). Primers used to amplify the coding and flanking non-coding regions of TUBA1A were designed using Primer 3 (TUBA1A ex3F: 5’-CTGGTCACTCACCCACTC-3’, TUBA1A ex3R: 5’-AACAGTTCAATTCTGTGTTTG-3’). Double-stranded DNA sequence analysis was performed using the Big Dye Terminator chemistry (Applied Biosystems, Foster City, CA, USA), and reactions were run on the ABI 3730_l Genetic Analyzer (Applied Biosystems). Sequence chromatograms were analyzed using Mutation Surveyor software version 3.30. Sequences were compared with normal control samples and the reference sequences for *TUBA1A*.

**Figure 3:**
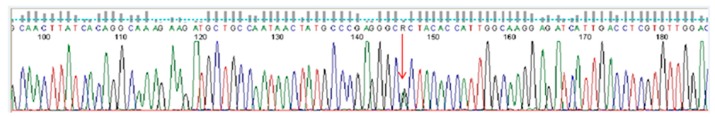
Partial electropherogram of the Sanger sequencing of *TUBA1A* in the patient shows (red arrow) the c.320A>G mutation.

A heterozygous c.320A>G [p.(His 107Arg) variant was identified in exon 2 of the *TUBA1A* gene (GenBank Accession: NM_006009.3) and confirmed by Sanger methods (Figure 3). The variant had de novo origin because it was not detected in the parents. There were no pathogenic mutations related to malformations of cortical development in any of the other 79 genes in the panel. The variant c.320A>G was not found in ExAC or the 1000 Genome database, and it was predicted to be deleterious by two prediction tools (MutationTaster, SNPdryad), whereas a third prediction software program (PolyPhen2) indicated the variant as probably benign. It is noteworthy that this variant substitutes a highly conserved amino acid histidine with an arginine in the N-terminal domain of the tubulin protein, which contains the guanine nucleotide-binding region and has GTPase activity, where many other pathogenic *TUBA1A* mutations are distributed.

## 3. Discussion

This case shows a patient with a de novo heterozygous c.320A>G [p.(His 107 Arg)] mutation in the *TUBA1A* gene that expands our knowledge on the spectrum of phenotypes associated with *TUBA1A* mutation. Disorders of microtubule formation and function and alterations of microtube-associated proteins are typically linked to a series of central nervous system defects. An impairment of mitosis leads to microcephaly. Undermigration or overmigration of neuronal cells causes cerebral cortical dysgenesis. Impaired axonal navigation is associated with anomalies of corpus callosum and cranial nerves. Alterations in the formation of the internal capsule lead to cortical dysgyria. Finally, a combination of defects in cell migration and axonal navigation is the reason for asymmetric brainstem and modified cerebellar vermis. Clinically, these deep modifications of the central nervous system structure lead to various neurological manifestations that depend on the degree of impairment of the different steps of development. Regarding the role played by genetic alterations of the various genes involved in the determination of microtubule structure and function, *TUBB2B* gene mutations have been associated with various types of lissencephaly and axon dysinnervation [13,14]. Defects in the *TUBB5* gene have been described in individuals with a simplified gyral pattern and early-onset epileptic seizures [15]. The core phenotype of *TUBA1A*-related tubulinopathies consists of lissencephaly, most frequently classic or with cerebellar hypoplasia [5]. Initial reports focused on the presence of other specific abnormalities found in *TUBA1A*-related lissencephalies, and these consist of a unique combination of microcephaly, pachygyria, complete or partial agenesis of the corpus callosum, cerebellar hypoplasia involving mainly the inferior vermis, brain stem hypoplasia, disorganization of the hippocampus, and dysmorphism of the basal ganglia [16]. The presence in our patient of this very peculiar pattern of congenital defects, together with dysmorphism of the basal ganglia (i.e., a pathognomonic feature of tubulinopathies), suggests a pathogenetic role of the rare de novo c.320A>G p. (p.His107Arg) variant identified. 

The patient here described showed not only complex brain anomalies (i.e., pachygyria, thin corpus callosum, hypoplastic basal ganglia) but also microcephaly, severe developmental delay, absent speech, and poorly controlled epilepsy, putting this phenotype among the severe forms of tubulinopathies. Bahi-Buisson et al. observed that severe tubulinopathies were more often associated with tubulin mutations affecting the GTP binding pocket, although these mutations were never found in milder phenotypes [5]. Our report reinforces this genotype-phenotype correlation, since the p.His107Arg variant affects the GTPase domain of the *TUBA1A* protein. However, the position of the mutations in the protein domains is not the only discrimination in the genotype-phenotype correlation. For example, it is known that the substitution p.R264C is invariably associated with central pachygyria, whereas the p.R264H mutation that affects the same amino acid residue causes microlissencephaly with complete agenesis of the corpus callosum, one of the most severe forms of tubulinopathy [5]. Similarly, the consequences of recurrent substitutions p.R422H and p.R422C are distinguishable because the former is responsible for lissencephaly, whereas p.R422C causes central pachygyria [17]. On the basis of this observation, it is not predictable whether the phenotype is related to other potential missense variants affecting the 107 residue, but it does appear to play a critical role for the correct functioning of the *TUBA1A* protein. Regarding the inheritance model, more than 95% of individuals diagnosed with a tubulinopathy have a de novo pathogenic variant. Rarely, an individual diagnosed with a tubulinopathy has an affected parent. In these few families, mutations in either *TUBB3* or *TUBB2B* genes have been identified. However, Jansen et al. [18] reported two sisters with perisylvian polymicrogyria, gray matter heterotopia, and enlarged lateral ventricles. Both sisters had the same heterozygous mutation in the *TUBA1A* gene [c.13A>C (p.Ile5Leu)] inherited from their mother, who was somatic mosaic for the mutation, which was found in 5.6% of her peripheral blood. An MRI of the clinically asymptomatic mother showed a thin corpus callosum, hypoplasia of the superior vermis, and a thin medulla. This report indicates that rare familial recurrence of *TUBA1A* related cerebral malformation can occur, with subclinical effect in the presence of mosaic state. While this situation is unusual, several groups have reported somatic pathogenic variants in genes encoding tubulin [19,20].

## 4. Conclusions

*TUBA1A* mutations lead to a variety of brain malformations ranging from lissencephaly with perisylvian pachygyria to diffuse posteriorly predominant pachygyria, combined with internal capsule dysgenesis, cerebellar dysplasia, and callosal hypotrophy. This peculiar MRI pattern, in combination with the usually severe clinical presentation, suggests the need for molecular studies to determine the mechanism of *TUBA1A* mutation-induced pathology.

## Supplementary Materials

Supplementary materials can be found at http://www.mdpi.com/1422-0067/18/11/2273/s1.
